# Application and prospect analysis of chimeric antigen receptor T-cell therapy in hepatocellular carcinoma treatment: a systematic review and meta-analysis

**DOI:** 10.3389/fimmu.2025.1566976

**Published:** 2025-04-07

**Authors:** Sichang Wu, Xinli Gan, Shuxin Huang, Yujun Zhong, Jialin Wu, Haojie Yang, Bangde Xiang

**Affiliations:** ^1^ Department of Hepatobiliary Surgery, Guangxi Medical University Cancer Hospital, Nanning, China; ^2^ The Second Clinical Medical College of Guangxi Medical University, Nanning, China; ^3^ Department of Hepatobiliary Surgery, Changde Hospital (The First People’s Hospital of Changde), Xiangya School of Medicine, Central South University, Changde, China

**Keywords:** CAR-T therapy, hepatocellular carcinoma, immunotherapy, meta-analysis, systematic review

## Abstract

**Introduction:**

Hepatocellular carcinoma (HCC), accounting for 90% of primary liver cancers, is a high-mortality malignancy and the third leading cause of cancer-related deaths globally, with major risk factors like hepatitis B/C, aflatoxin exposure, and obesity. Most patients are diagnosed at advanced stages, with a 5-year survival rate below 10%. Therefore, HCC treatment and research still face significant challenges, and more effective treatments need to be further explored.

**Methods:**

We searched PubMed, Scopus, Web of Science and Embase from the time of repository construction to March 1, 2025, preliminary included studies involving animal experiments on the therapeutic effects of Chimeric Antigen Receptor T-cell (CAR-T cell) therapy on HCC. After exclusion and evaluation of literature, the random/fixed effects model was employed to perform meta-analysis and obtain Weighted Mean Difference (WMD) and 95% confidence interval (CI) of tumor volume and mass. We then verify the robustness of the results through subgroup analysis and sensitivity analysis. Use Q-test to evaluate heterogeneity and quantify it based on *I²* value.

**Results:**

We included a total of 16 studies. Multiple independent sets of data were extracted from the experiments of these studies, of which 25 were used for volume-based meta-analysis and 16 were used for mass-based meta-analysis. Regarding volume, The combined mean CAR-T treatment group/control group resulted in an WMD of -515.77 (95% CI: -634.78 to -396.76; *I²* =90.8%). Meanwhile, based on mass, the combined mean CAR-T treatment group/control group resulted in an WMD of -0.30 (95% CI: -0.38 to -0.22; *I²* = 94.4%). The results of the bias analysis further validated the reliability of the research conclusions.

**Conclusions:**

Based on the dual-index meta-analysis, the CAR-T therapy have been proved to possess significant therapeutic effect in HCC. However, the funnel plot of tumor mass and the Egger’s regression suggest the potential presence of publication bias. Thus, it warrants further research to evaluate the potential of CAR-T therapy alone or as an adjuvant for HCC treatment.

## Introduction

Hepatocellular carcinoma (HCC) ranks as the sixth most prevalent cancer globally and represents the third leading cause of cancer-related mortality (1). China bears the highest global burden of HCC cases, where it stands as the second most prevalent tumor and exhibits the highest mortality rate. The management of HCC is contingent upon tumor size and location, the patient’s overall health status, and the presence of cirrhosis. Therapeutic strategies encompass surgical resection, liver transplantation, local ablative therapies (e.g., radiofrequency ablation, microwave ablation), transarterial chemoembolization (TACE), targeted therapy, and immunotherapy (2). However, these approaches still have some limitations in the treatment of HCC, and existing treatments such as surgical resection, chemotherapy and targeted therapy have significant limitations in terms of efficacy and patient survival. Surgical resection is only suitable for early-stage patients and has a high recurrence rate, while chemotherapy and targeted therapy are often accompanied by serious side effects and drug resistance problems. According to the Barcelona Clinic Liver Cancer (BCLC) staging system (3), patients with very early (stage 0) and early (stage A) HCC are candidates for surgical resection, ablation, or liver transplantation; however, for those with advanced HCC, these modalities are no longer recommended. Advanced HCC typically involves extensive liver involvement, vascular infiltration, and distant metastases, rendering its treatment particularly challenging and necessitating the exploration of superior therapeutic strategies. Consequently, the development of novel therapeutic strategies is imperative.

Chimeric Antigen Receptor T-cell (CAR-T cell) therapy is a clinically effective and target-specific immunotherapeutic approach that involves genetically engineering a patient’s T cells to express a chimeric antigen receptor (CAR), enabling the specific recognition and elimination of tumor cells. This precise targeting mechanism allows CAR-T therapy to circumvent the issues of non-specific toxicity and drug resistance commonly associated with conventional therapies, including chemotherapy and radiotherapy. To date, CAR-T therapies have demonstrated remarkable success in the treatment of hematologic malignancies. For instance, CD19-targeted CAR-T cell therapies have achieved high complete remission rates in patients with relapsed or refractory B-cell acute lymphoblastic leukemia (B-ALL) and diffuse large B-cell lymphoma (DLBCL) (4). In 2017, the U.S. FDA approved the first CAR-T therapy for B-ALL, representing a significant milestone in the clinical application of CAR-T therapy (5). Additionally, BCMA-targeted CAR-T therapies have exhibited notable efficacy in multiple myeloma, offering valuable insights for the treatment of solid tumors (6, 7). The biological characteristics of HCC render it a potentially ideal candidate for CAR-T therapy. HCC is characterized by a highly heterogeneous and immunosuppressive tumor microenvironment (TME), which significantly restricts the efficacy of conventional therapies, including chemotherapy and targeted therapies. However, CAR-T therapy can overcome the immunosuppressive nature of the tumor microenvironment and achieve precise tumor eradication by genetically engineering T cells to specifically target HCC-associated antigens, such as Glypican-3 (GPC3), alpha-fetoprotein (AFP), and phosphatidylinositol proteoglycan-3. GPC3 is overexpressed in 70%-80% of HCC cases but is virtually absent in normal tissues, making it an ideal target for CAR-T therapy (8). Recent clinical and preclinical studies have highlighted the remarkable efficacy of CAR-T cells in targeting HCC, with several molecules, including GPC3, AFP, c-Met, and CD133, emerging as promising targets for immunotherapy in HCC (9). For instance, Batra et al. demonstrated that GPC3-CAR T cells co-expressing IL15 and IL21 exhibited significant efficacy in preclinical models of HCC (10). Conversely, Zhou et al. reported that bispecific CAR-T cells demonstrated superior antitumor efficacy compared to single-targeted CAR-T cells in both *in vitro* and *in vivo* settings, potentially offering novel therapeutic strategies for HCC patients and mitigating tumor recurrence due to antigenic heterogeneity (11). Furthermore, AFP-targeted CAR-T cells exhibited significant anti-HCC activity in both *in vivo* and *in vitro* studies (12), while high CD133 expression in HCC was associated with poor prognosis (13).

Given the significant potential of CAR-T therapy in HCC, this study aims to systematically evaluate its therapeutic efficacy and elucidate its underlying mechanisms through preclinical experiments. Our objectives include providing a robust theoretical foundation and empirical data to support future clinical trials, optimizing CAR-T cell design to counteract tumor microenvironment suppression, and exploring combination treatment strategies. These efforts are expected to advance the clinical application of CAR-T therapy in HCC treatment.

## Methods

### Literature search

Two researchers independently performed a systematic literature search across four major academic databases: PubMed, Embase, Web of Science, and Scopus. Multiple keyword combinations related to CAR-T therapy and HCC were employed during the search process to maximize the retrieval of relevant studies. These keyword combinations encompassed not only core terms (e.g., “CAR-T” and “HCC”) but also related synonyms, variations, and potential research directions (e.g., “chimeric antigen receptor T cell”, “hepatocellular carcinoma”). Terms such as “hepatocellular carcinoma” and “immunotherapy” were included to minimize the risk of omitting relevant studies. The search timeframe spanned from the inception of each database to March 1, 2025. Following the completion of the searches, the two researchers independently organized and compared their respective search results. In cases of discrepancies, the researchers re-evaluated their search steps, strategies, and database settings to ensure the accuracy and consistency of the results. The detailed search formulas utilized by the researchers are presented below. The search formulas were tailored to each database’s specific syntax and field requirements to accommodate their unique characteristics.

#### Pubmed

((“CAR-T cell therapy” OR “chimeric antigen receptor T cell therapy” OR “CAR-T” OR “CAR-T cells” OR “CAR T cell therapy” OR “chimeric antigen receptor T-cells” OR “CAR-T immunotherapy” OR “chimeric antigen receptor T-cell therapy” OR “CAR T-cell immunotherapy” OR “chimeric antigen receptor therapy” OR “CAR-based immunotherapy” OR “CAR-engineered T cells” OR “chimeric antigen receptor-modified T cells” OR “CAR modified T-cells” OR “CAR redirected T cells” OR “genetically engineered T cells” OR “adoptive T-cell therapy” OR “CAR-modified immune cells”) AND (“liver cancer” OR “hepatocellular carcinoma” OR “hepatoma” OR “liver neoplasm” OR “liver cell carcinoma” OR “hepatic cancer” OR “HCC” OR “primary liver cancer” OR “liver malignancy” OR “hepatic tumor” OR “liver carcinoma” OR “liver tumor” OR “hepatic cell carcinoma” OR “hepatic neoplasm” OR “hepatocarcinoma” OR “hepatocyte carcinoma” OR “primary hepatic cancer” OR “liver lesion”))

#### Embase

(‘CAR-T cell therapy’/exp OR ‘CAR-T cell therapy’ OR ‘chimeric antigen receptor T cell therapy’/exp OR ‘chimeric antigen receptor T cell therapy’ OR ‘CAR-T’/exp OR ‘CAR-T’ OR ‘CAR-T cells’/exp OR ‘CAR-T cells’) AND (‘liver cancer’/exp OR ‘liver cancer’ OR ‘hepatocellular carcinoma’/exp OR ‘hepatocellular carcinoma’ OR ‘hepatoma’/exp OR ‘hepatoma’ OR ‘liver neoplasm’/exp OR ‘liver neoplasm’ OR ‘liver cell carcinoma’/exp OR ‘liver cell carcinoma’ OR ‘hepatic cancer’/exp OR ‘hepatic cancer’)

#### Web of Science

TS=((“CAR-T cell* therap*” OR “chimeric antigen receptor T cell* therap*” OR “CAR-T” OR “CAR-T cell*” OR “CAR T cell* therap*” OR “chimeric antigen receptor T-cell*” OR “CAR-T immunotherap*” OR “chimeric antigen receptor T-cell* therap*” OR “CAR T-cell* immunotherap*” OR “chimeric antigen receptor therap*” OR “CAR-based immunotherap*” OR “CAR-engineered T cell*” OR “chimeric antigen receptor-modified T cell*” OR “CAR modified T-cell*” OR “CAR redirected T cell*” OR “genetically engineered T cell*” OR “adoptive T-cell* therap*” OR “CAR-modified immune cell*”) AND (“liver cancer” OR “hepatocellular carcinoma” OR “hepatoma” OR “liver neoplasm*” OR “liver cell carcinoma” OR “hepatic cancer” OR “HCC” OR “primary liver cancer” OR “liver malignancy” OR “hepatic tumor*” OR “liver carcinoma” OR “liver tumor*” OR “hepatic cell carcinoma” OR “hepatic neoplasm*” OR “hepatocarcinoma” OR “hepatocyte carcinoma” OR “primary hepatic cancer” OR “liver lesion*”))

#### Scopus

(TITLE-ABS-KEY(“CAR-T cell therapy”) OR TITLE-ABS-KEY(“chimeric antigen receptor T cell therapy”) OR TITLE-ABS-KEY(“CAR-T”) OR TITLE-ABS-KEY(“CAR-T cells”) OR TITLE-ABS-KEY(“CAR T cell therapy”) OR TITLE-ABS-KEY(“chimeric antigen receptor T-cells”) OR TITLE-ABS-KEY(“CAR-T immunotherapy”) OR TITLE-ABS-KEY(“chimeric antigen receptor T-cell therapy”) OR TITLE-ABS-KEY(“CAR T-cell immunotherapy”) OR TITLE-ABS-KEY(“chimeric antigen receptor therapy”) OR TITLE-ABS-KEY(“CAR-based immunotherapy”) OR TITLE-ABS-KEY(“CAR-engineered T cells”) OR TITLE-ABS-KEY(“chimeric antigen receptor-modified T cells”) OR TITLE-ABS-KEY(“CAR modified T-cells”) OR TITLE-ABS-KEY(“CAR redirected T cells”) OR TITLE-ABS-KEY(“genetically engineered T cells”) OR TITLE-ABS-KEY(“adoptive T-cell therapy”) OR TITLE-ABS-KEY(“CAR-modified immune cells”)) AND (TITLE-ABS-KEY(“liver cancer”) OR TITLE-ABS-KEY(“hepatocellular carcinoma”) OR TITLE-ABS-KEY(“hepatoma”) OR TITLE-ABS-KEY(“liver neoplasm”) OR TITLE-ABS-KEY(“liver cell carcinoma”) OR TITLE-ABS-KEY(“hepatic cancer”) OR TITLE-ABS-KEY(“HCC”) OR TITLE-ABS-KEY(“primary liver cancer”) OR TITLE-ABS-KEY(“liver malignancy”) OR TITLE-ABS-KEY(“hepatic tumor”) OR TITLE-ABS-KEY(“liver carcinoma”) OR TITLE-ABS-KEY(“liver tumor”) OR TITLE-ABS-KEY(“hepatic cell carcinoma”) OR TITLE-ABS-KEY(“hepatic neoplasm”) OR TITLE-ABS-KEY(“hepatocarcinoma”) OR TITLE-ABS-KEY(“hepatocyte carcinoma”) OR TITLE-ABS-KEY(“primary hepatic cancer”) OR TITLE-ABS-KEY(“liver lesion”))

### Inclusion and exclusion criteria

In this study, two researchers independently screened articles based on their titles, abstracts, and full texts. Any discrepancies between the two researchers were resolved through discussion to reach a consensus. If consensus could not be reached, a third senior researcher made the final decision following a group discussion. Initially, the researchers reviewed the titles and abstracts of all articles and selected studies that met the following criteria:

#### Inclusion criteria

- The study focused on animal models specifically designed to evaluate the therapeutic efficacy of CAR-T cells.
- The study investigated the therapeutic application of CAR-T therapy.
- The study included a control group for comparative analysis.
- The study reported efficacy-related outcomes, including tumor volume and tumor mass, among other relevant parameters.
- The study was designed as an experimental or controlled trial.

#### Exclusion criteria

- Studies not involving animal experiments, such as clinical trials or *in vitro* studies, were excluded.
- Studies unrelated to CAR-T cell therapy were excluded.
- Studies lacking a control group or with ineligible control groups, such as those with only a single treatment arm, were excluded.
- Studies failing to report relevant efficacy outcomes were excluded.
- Studies failing to report relevant efficacy outcomes were excluded.
- Studies with designs that did not meet the criteria for experimental or controlled trials were excluded.

### Subsequently, full-text screening was conducted to exclude the following articles

#### Inclusion Criteria

- The animal models used must be clearly specified, with detailed information provided regarding their source and characteristics. All experimental animals underwent rigorous screening prior to the study to ensure the absence of pre-existing diseases and HCC-related genetic mutations. Additionally, the study must adhere to relevant ethical standards.
- The type of CAR-T cells used must be clearly described, along with detailed construction information, including the design of the CAR. The treatment protocol for CAR-T cells, including the administration route and dosage, must be explicitly outlined. Quality control standards for CAR-T cells, such as cell purity, viability, and transduction efficiency, should be thoroughly documented.
- The control groups must be clearly defined, including types such as blank controls (no treatment) and traditional treatment controls. Detailed information on control group treatments, such as administration routes and dosages, as well as baseline characteristics of control animals (e.g., strain and cell lines used for model construction), should be provided.
- Efficacy outcomes must be reported in detail, including descriptions of tumor volume and mass measurements, the tools used for measurement, and quantitative data on tumor volume changes at specific time points (e.g., 1 week and 4 weeks post-treatment).
- The study design framework must be clearly described, including detailed standards for animal experiments and laboratory quality control measures. The design of control and experimental groups should ensure rationality, and all experimental data must be fully recorded and analyzed using appropriate statistical methods to ensure scientific rigor and credibility.

### The literature quality assessment

To ensure methodological rigor, two independent researchers systematically evaluated all included studies using both the Cochrane Risk of Bias Tool and the Systematic Evaluation Center for Laboratory Animal Studies (SYRCLE) Risk of Bias Tool. In cases of disagreement, a third senior researcher facilitated a consensus discussion, with final decisions based on comprehensive evaluation of all available evidence. The risk of bias assessment encompassed critical domains such as randomization methodology (including sequence generation and allocation concealment), blinding procedures for both researchers and outcome assessors, management of incomplete outcome data, selective outcome reporting, study design appropriateness, statistical methodology, and other potential sources of bias. The exclusion criteria were strictly applied to studies demonstrating high risk of bias in critical domains (particularly regarding randomization procedures or blinding implementation), studies with attrition rates exceeding 20% without appropriate statistical handling of missing data, and studies lacking documentation of randomization or blinding procedures. Furthermore, studies classified as having a “high” overall risk of bias or containing irremediable methodological flaws were systematically excluded from the final analysis. When potential biases could not be adequately mitigated through sensitivity analyses or subgroup stratification, such studies were consequently excluded to maintain the methodological integrity and reliability of the meta-analytic findings.

### Data extraction and processing

Data extraction was independently performed by two reviewers, and the final data results were calculated as the average of the values extracted by both reviewers. If the discrepancy exceeded 10%, resolution was achieved through discussion or consultation with a third senior reviewer to ensure data consistency and accuracy. The extracted data were categorized into two main groups: study characteristics (including author, publication year, country, sample size, animal model, and CAR-T cell type) and outcome metrics (such as tumor mass and tumor volume). For studies where raw data were not directly available, numerical data were extracted from graphical representations using WebPlotDigitizer software (version 4.2), while data lacking reliable variance information were excluded. When essential raw data (such as mean values, standard deviations, or sample sizes) were not reported but graphical representations (e.g., bar graphs, line graphs, or scatter plots) were available, WebPlotDigitizer was employed for data extraction to supplement the missing statistical information. In cases where studies provided partial statistical parameters (such as mean values without corresponding standard deviations), graphical data extraction was utilized to obtain the missing values. Additionally, when discrepancies were identified between reported numerical data and graphical representations, data extracted from figures using WebPlotDigitizer were used to verify the accuracy of the reported values.

### Statistical analysis and bias detection

Statistical analyses were performed using Stata software (version 18.0). The weighted mean difference (WMD) was employed as the effect size measure to pool continuous data across studies. WMD is an appropriate effect size metric for studies with identical measurement units, as it weights the mean differences from individual studies by their sample sizes, thereby minimizing the influence of sample size variability. For WMD calculation, the effect size for each study was derived by subtracting the control group mean from the experimental group mean, with results expressed in their original measurement units for straightforward interpretation. Heterogeneity was assessed using the *I²* statistic and Cochran’s Q test. The *I²* statistic quantifies the proportion of between-study variability, calculated as *I²* = 100% × (Q - df)/Q, where Q represents Cochran’s Q statistic and df denotes the degrees of freedom. Based on the *I²* value and the P-value from Cochran’s Q test, the appropriate statistical model was selected: a fixed-effects model was applied when *I²* < 50% and P > 0.1, indicating low heterogeneity, whereas a random-effects model was used when *I²* ≥ 50% and P ≤ 0.1, accounting for significant between-study variability. The fixed-effects model assumes that all studies share a common true effect size, whereas the random-effects model allows for variability in true effect sizes across studies. To explore potential sources of heterogeneity, subgroup analyses were conducted based on the following factors: mice strain, tumor burden, cell lines used for model construction, and the country of study origin. Additionally, sensitivity analysis was performed using the leave-one-out method to evaluate the influence of individual studies on the overall effect size. If the exclusion of a study resulted in substantial changes in the effect size, the study was considered influential and further examined for potential biases. Finally, publication bias was assessed using funnel plots. If significant asymmetry was observed in the funnel plots, Egger’s regression was employed to complement visual inspection and quantify potential bias.

## Results

### Inclusion of literature and general information on the study

**Figure 1:**
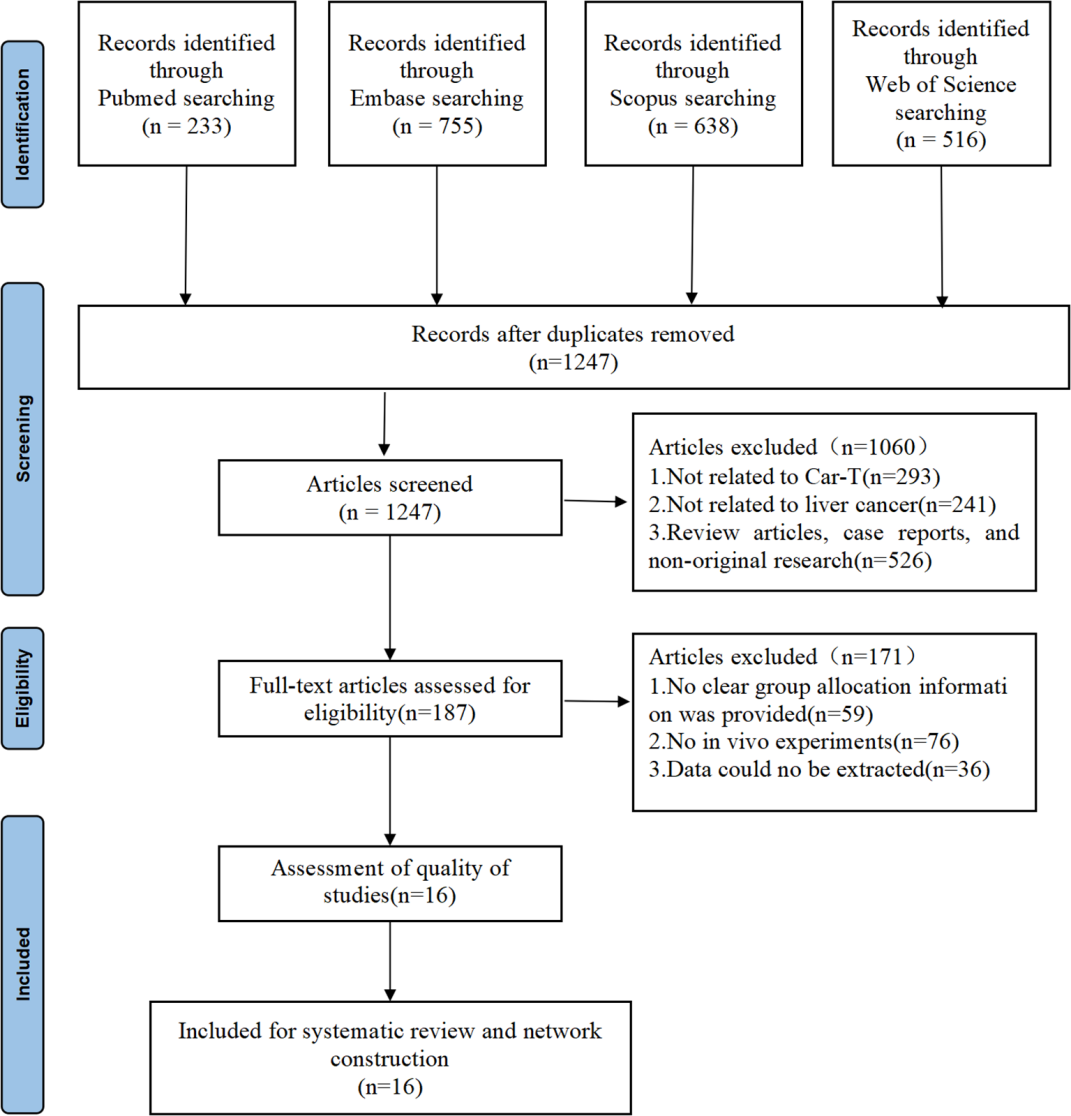
PRISMA flow diagram.

As shown in **Figure 1**, preliminary searches in PubMed and Embase identified 233 and 755 relevant articles, respectively. Additionally, searches in Scopus and Web of Science yielded 638 and 516 articles, respectively. After deduplication and screening, a total of 16 studies (14–28) were ultimately included for further investigation of the therapeutic effects of CAR-T on HCC. Among the 16 included studies (**Table 1**), the majority were conducted in China (n=13) and the United States (n=3). The experimental animals primarily consisted of immunodeficient mice models, such as NOD/SCID, NSG, and NCG mice, with a small proportion of immunocompetent mice models (e.g., C57BL/6 mice). The age of the animals ranged from 4 to 8 weeks. Commonly used HCC cell lines in these experiments included HepG2, Huh7, Hep3B, and PLC/PRF/5. The intervention groups were treated with various types of CAR-T cells (e.g., GPC3-CAR T cells, CD147-CAR-T cells), while the control groups received PBS, untransduced (UTD) T cells, or saline. The sample sizes ranged from 4 to 10 animals per group, with most studies maintaining equal numbers in the experimental and control groups. The diversity in experimental designs, animal models, and intervention strategies across the included studies provides a robust foundation for this meta-analysis.

**Table 1:** Characteristics of studies included in the meta-analysis.

Author	Publication year	Country	Animal species	Cell lines	Age of animal	Sample size		Intervention	
						E	C	E	C
Zhang et al. (26)	2024	China	NOD/SCID mice	Huh7/HepG2	6-8w	5	5	CAR-T cells	0.9 % saline
Zou et al. (28)	2024	China	NSG mice	Hep3B	6-7w	5	5	B7-H3 CAR-T cells	PBS
Sun et al. (22)	2022	China	C57BL/6 mice NPG mice	E0771-GPC3 PLC/PRF/5	NA	5	5	28ζ and BBζ CAR T cells m-28ζ and m-BBζ CAR T cells	UTD T cells
Liu et al. (12)	2017	America	SCID-Beige mice	HepG2	6-8w	6/8	6/8	AFP-CAR T cells	Mock T cells
Guo et al. (16)	2018	China	NSG mice	PLC/PRF/5	6-8w	7	7	PD-1-deficient GPC3-CAR T cells wild-type GPC3-CAR T cells	UTD T cells
Wu et al. (25)	2019	China	C57BL/6 mice	Hepa1-6-chGPC3	4-6w	5/6	5/6	GPC3-CAR T cells	UTD T cells/Vehicle
Zhang et al. (27)	2019	China	Nude mice	Huh7	6-8w	6	6	Tet-CD147CART cells	PBMCs
Tseng et al. (23)	2020	America	NSG mice	SK-Hep1	8w	10	10	CD147-CAR-T	PBS
Sun et al. (20)	2021	China	NCG mice	Hep3B	4-6w	7	7	hYP7 CAR-T cells 32A9 CAR-T cells CD19 CAR-T cells	PBS
Huang et al. (17)	2021	China	BALB/c nude mice	HepG2	4-5w	6	6	c-Met-28-3ζ CAR-T cells c-Met-137-3ζ CAR-T cells c-Met-28-137-3ζ CAR-T cells	Activated T cells
Torchia et al. (15)	2022	America	NSG mice	Hep3B	6-8w	10	10	CTR-CAR-T cells	UTD T cells
Sui et al. (19)	2025	China	NSG mice	Huh7	5-6w	7	7	NKBBz CAR-T cells GC3328z CAR-T cells GC33BBz CAR-T cells GC3328z-NKBB CAR-T cells	UTD T cells
Chen et al. (14)	2024	China	BALB/c nude mice	Hep3B	4-6w	5	5	GPC3-CAR T cells 21×9 GPC3 CAR-T cells	0.9 % saline
Lin et al. (18)	2024	China	NCG mice	Huh7	6-8w	4	4	Anti-FGFR4 Nb1 CAR-T cells Anti-FGFR4 Nb2 CAR-T cells	NC-T cells
Sun et al. (21)	2024	China	C57BL/6 mice	Hepa1-6-chGPC3	4-6w	5	5	h28Z CAR-T cells hAGK CAR-T cells hGLUT1 CAR-T cells	UTD T cells
Wang et al. (24)	2023	China	NOD/SCID mice	HepG2	6-8w	5	5	CD105 CAR T cells	UTD T cells

### Quality assessment of literature

The 16 studies were evaluated using the Cochrane Risk of Bias Tool and SYRCLE Risk of Bias Tool. Detailed evaluations of the quality of each study are presented in **Table 2**.

**Table 2:** Assessment of quality of studies.

Study(years)	Selection bias			Performance bias		Detection bias	Attrition bias	Reporting bias	Other
	Sequence generation	Baseline characteristics	Allocation concealment	Random housing	Blinding	Random outcome assessment	Incomplete outcome data	Selective outcome reporting	Other sources of bias
Zhang et al. (2024) (26)	Low risk	Unclear risk	Low risk	Low risk	Low risk	Low risk	Low risk	Low risk	Unclear risk
Zou et al. (2024) (28)	Low risk	Unclear risk	Low risk	Low risk	Unclear risk	Low risk	Low risk	Low risk	Unclear risk
Sun et al. (2022) (22)	Unclear risk	Unclear risk	Low risk	Low risk	Low risk	Low risk	Low risk	Unclear risk	Unclear risk
Liu et al. (2017) (12)	Unclear risk	Low risk	Low risk	Low risk	Low risk	Unclear risk	Low risk	Low risk	Unclear risk
Guo et al. (2018) (16)	Unclear risk	Unclear risk	Unclear risk	Low risk	Low risk	Low risk	Low risk	Low risk	Unclear risk
Wu et al. (2019) (25)	Low risk	Unclear risk	Low risk	Low risk	Low risk	Low risk	Low risk	Low risk	Unclear risk
Zhang et al. (2019) (27)	Low risk	Unclear risk	Low risk	Low risk	Low risk	Unclear risk	Low risk	Low risk	Unclear risk
Tseng et al. (2020) (23)	Unclear risk	Unclear risk	Low risk	Low risk	Unclear risk	Low risk	Low risk	Low risk	Unclear risk
Sun et al. (2021) (20)	Unclear risk	Unclear risk	Low risk	Low risk	Low risk	Unclear risk	Low risk	Low risk	Unclear risk
Huang et al. (2021) (17)	Low risk	Unclear risk	Unclear risk	Low risk	Low risk	Low risk	Low risk	Low risk	Unclear risk
Torchia et al. (2022) (15)	Low risk	Low risk	Low risk	Low risk	Low risk	Unclear risk	Low risk	Low risk	Unclear risk
Sui et al. (2025) (19)	Unclear risk	Unclear risk	Unclear risk	Low risk	Low risk	Unclear risk	Low risk	Low risk	Unclear risk
Chen et al.(2024) (14)	Low risk	Unclear risk	Low risk	Low risk	Low risk	Unclear risk	Low risk	Low risk	Unclear risk
Lin et al.(2024) (18)	Low risk	Low risk	Low risk	Low risk	Low risk	Unclear risk	Low risk	Low risk	Unclear risk
Sun et al.(2024) (21)	Low risk	Low risk	Low risk	Low risk	Low risk	Low risk	Low risk	Low risk	Unclear risk
Wang et al.(2023) (24)	Unclear risk	Low risk	Low risk	Low risk	Low risk	Unclear risk	Low risk	Low risk	Unclear risk

### Meta-analysis reveals significant therapeutic effects of CAR-T on HCC

**Figure 2:**
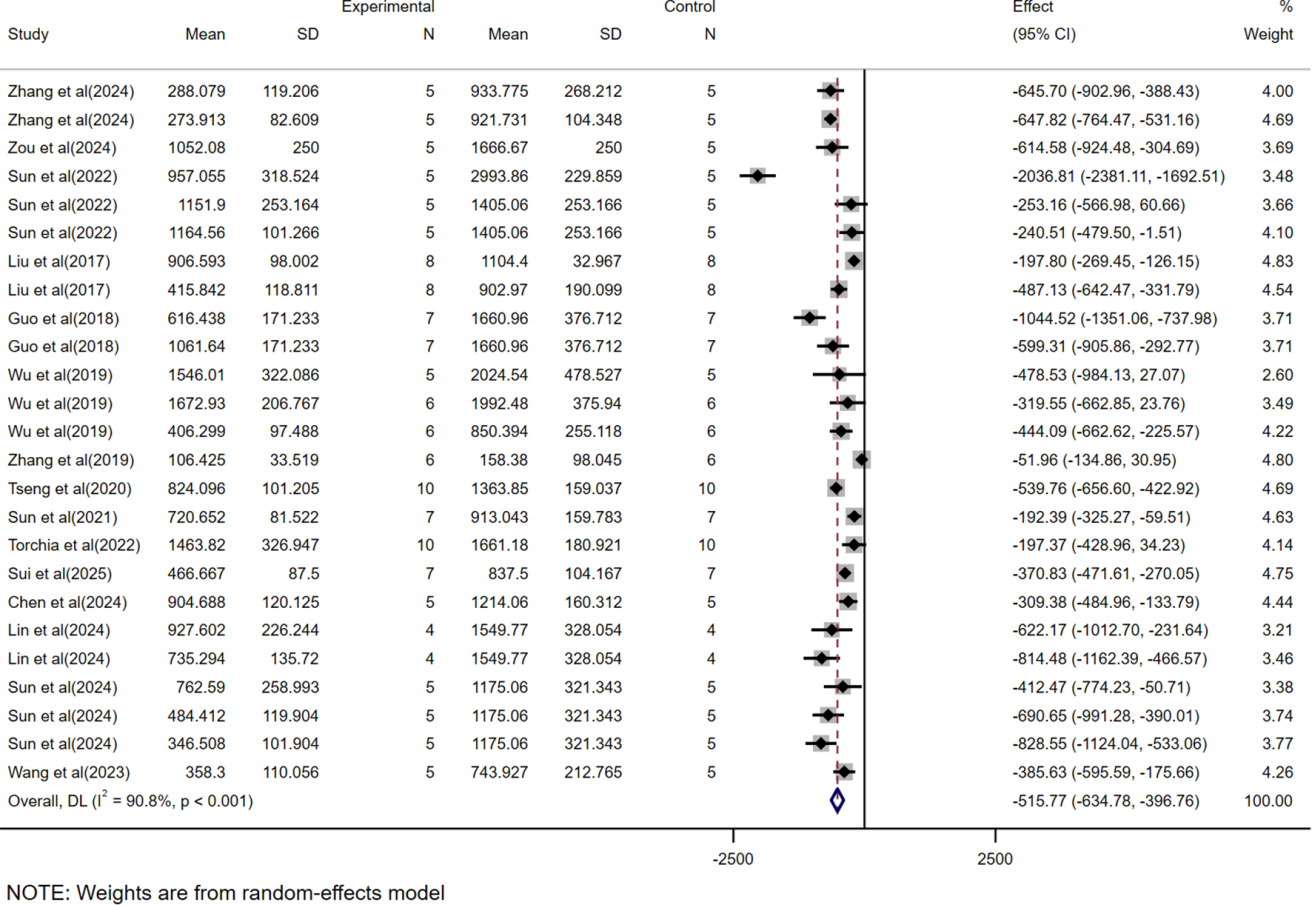
Meta-analysis forest plots based on tumor volume.

The WMD was used as the effect size because the scoring systems across studies were consistent, allowing direct comparison of means between groups. Among the 15 volume-based studies, multiple independent sets of data were extracted from a single experiment to validate the efficacy of CAR-T, as most of the studies included data from models constructed from multiple cell lines and different tumor burdens. A total of 25 volume-related datasets were obtained, showing an WMD of -515.77 (95% CI: -634.78 to -396.76) between the CAR-T treatment group and the control group, with high heterogeneity (*I²* =90.8%) (**Figure 2**).

**Figure 3:**
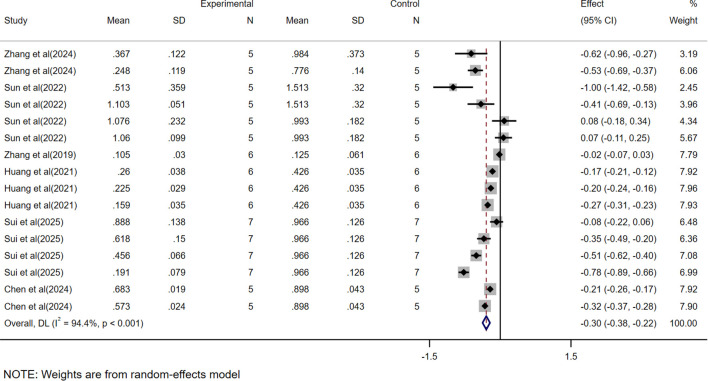
Meta-analysis forest plots based on tumor mass.

Regarding mass (**Figure 3**), the study included datasets from multiple cell lines and models constructed with different tumor burdens, and similarly, independent datasets could be extracted from each experiment to assess CAR-T efficacy. A total of 16 study datasets were collected, which showed a WMD of -0.30 (95% CI: -0.38 to -0.22) and high heterogeneity (*I²* = 94.4%). Therefore, to clarify the source of heterogeneity, we performed subgroup analyses.

### Subgroup analysis based on different experimental mice strains

**Figure 4:**
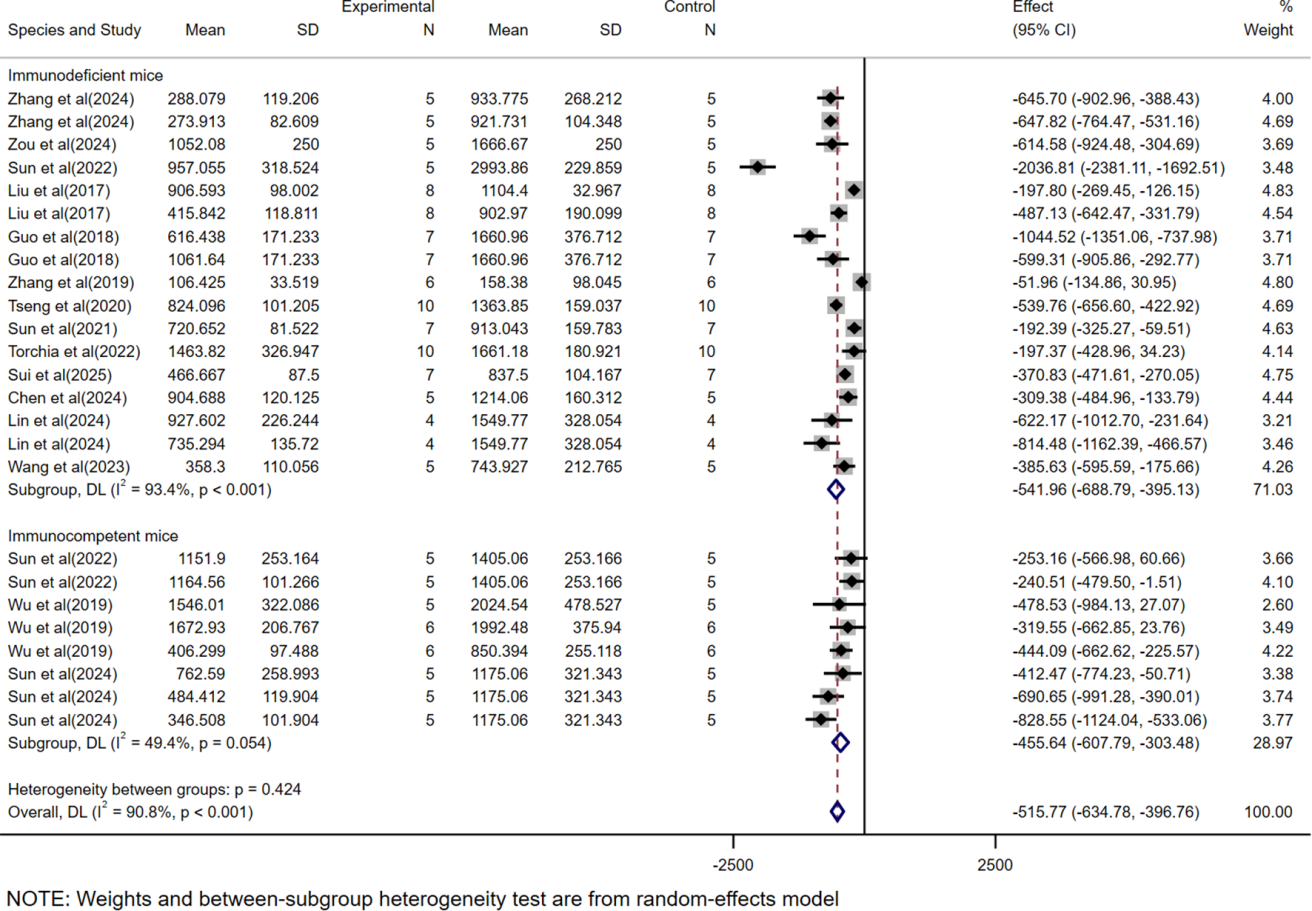
The subgroup analysis based on tumor volume according to experimental animal species.

Subgroup analysis based on tumor volume showed that CAR-T therapy was significantly effective in immunodeficient mice (WMD = -541.96, 95% CI: -688.79 to -395.13), although with high heterogeneity (*I²*= 93.4%). Similarly, in immunocompetent mice, the efficacy remained significant (WMD = -455.64, 95% CI: -607.79 to -303.48), but with Moderate heterogeneity (*I²*= 49.4%) (**Figure 4**).

**Figure 5:**
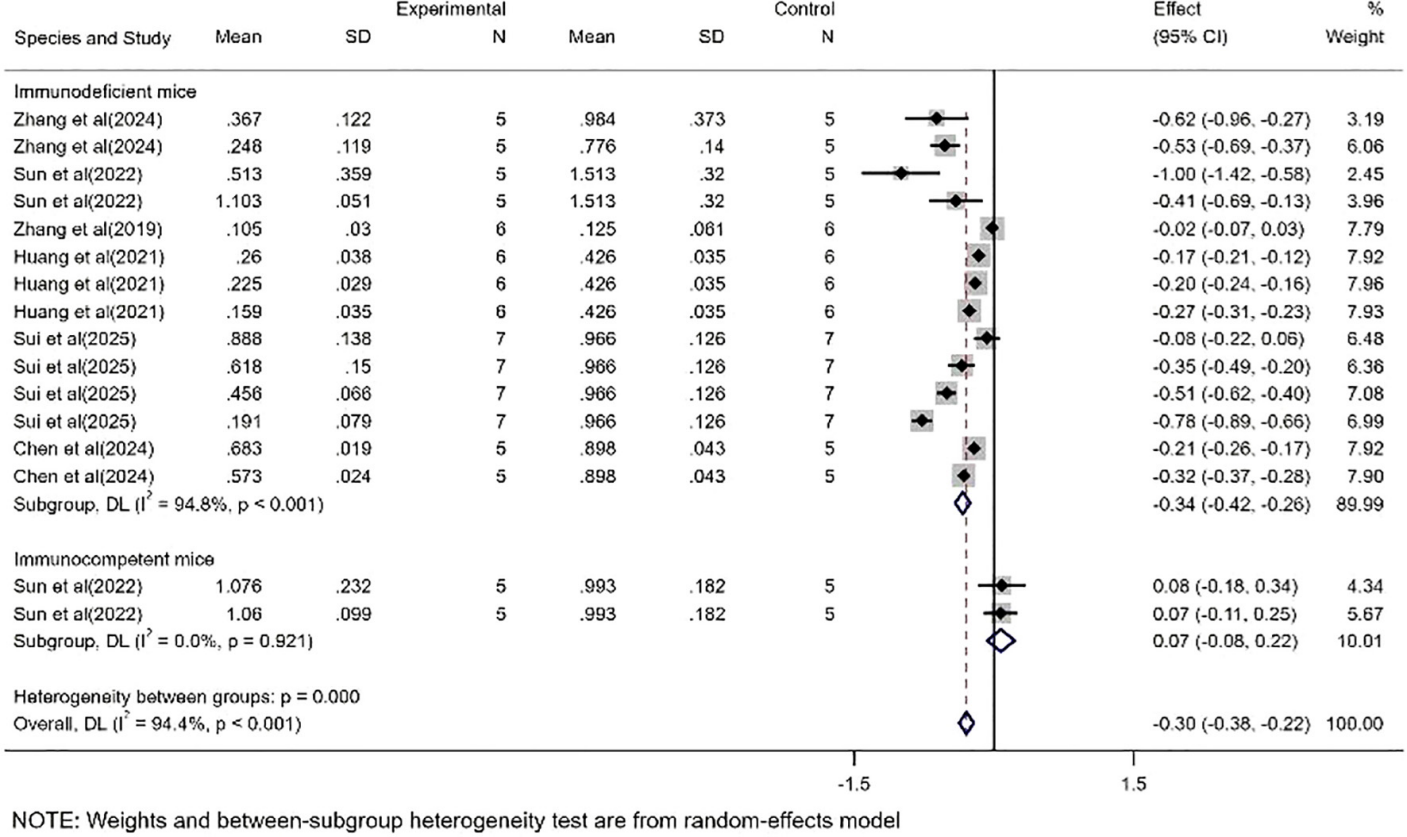
The subgroup analysis based on tumor mass according to experimental animal species.

Based on mass-related studies, CAR-T showed a significant therapeutic effect in the immunodeficient mice subgroup (WMD = -0.34, 95% CI: -0.42 to -0.26), with high heterogeneity (*I²* = 94.8%). However, no significant effect was observed in the immunocompetent mice subgroup (WMD = 0.07, 95% CI: -0.08 to 0.22) (**Figure 5**).

**Figure 6:**
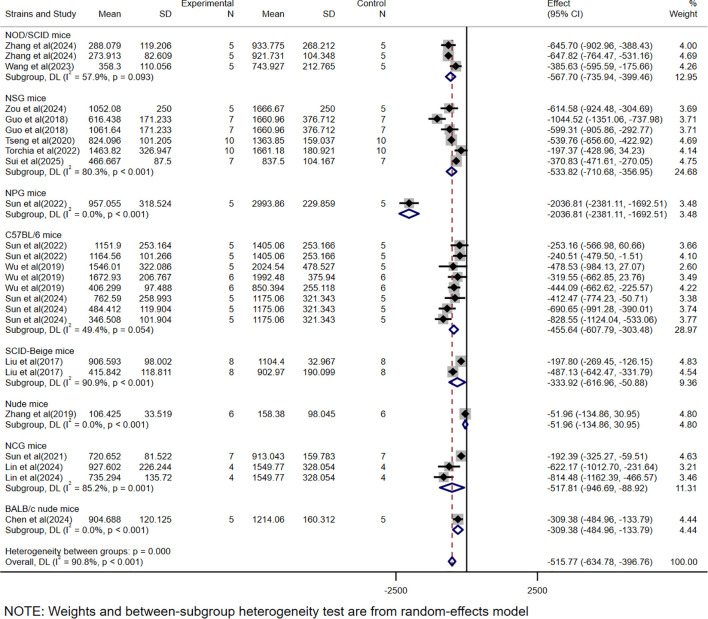
The subgroup analysis based on tumor volume according to experimental mice strains.

**Figure 7:**
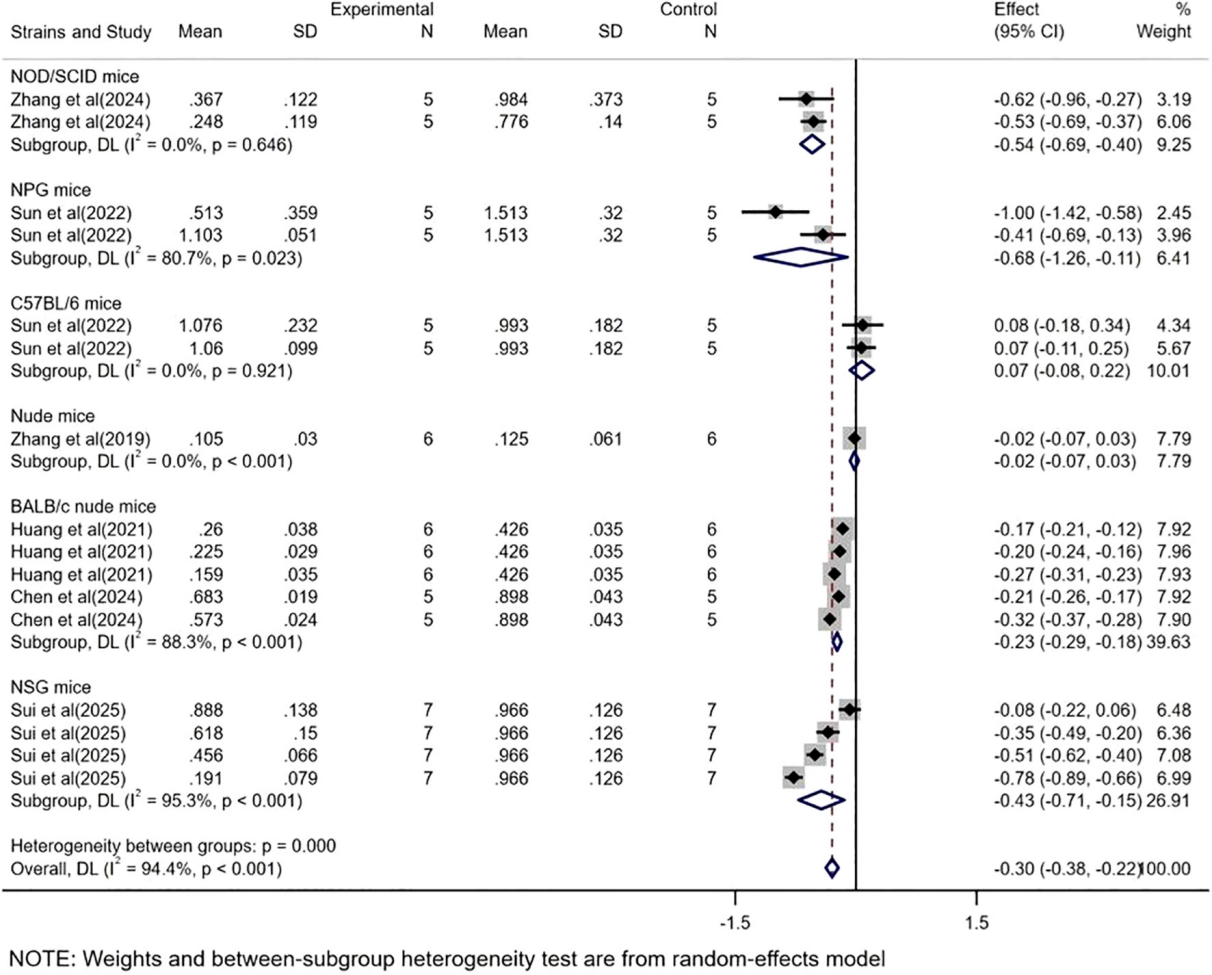
The subgroup analysis based on tumor mass according to experimental mice strains.

To further elucidate and control the high heterogeneity observed in the immunodeficient mice model, we conducted a subgroup analysis based on mice strains. The subgroup analysis based on tumor volume across different mice strains (**Figure 6**) demonstrated significant efficacy in all mice strain subgroups except for the Nude mice (WMD = -51.96, 95% CI: -134.86 to 30.95). Among the mice strains included in multiple studies, only C57BL/6 mice exhibited relatively low heterogeneity (*I²* = 49.4%). Furthermore, the subgroup analysis based on tumor mass across different mice strains (**Figure 7**) revealed significant efficacy in all subgroups except for the Nude mice (WMD = -0.02, 95% CI: -0.07 to 0.03) and C57BL/6 mice (WMD = 0.07, 95% CI: -0.08 to 0.22). Notably, the heterogeneity was consistently high across all mice strains included in multiple studies.

### Subgroup analysis based on different tumor burdens

**Figure 8:**
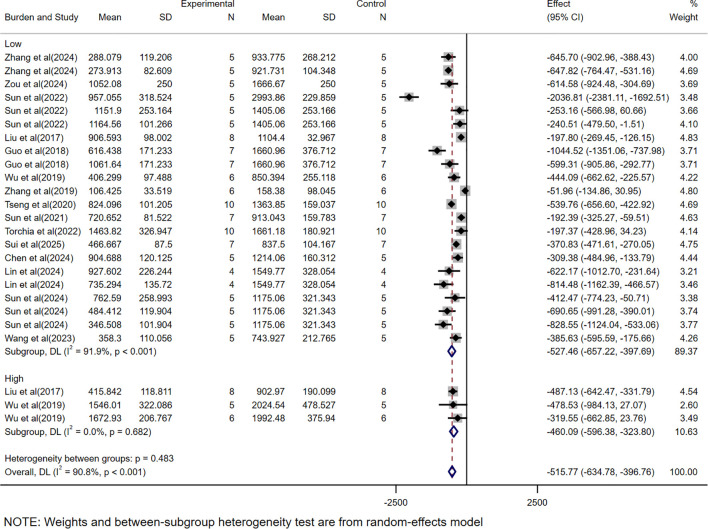
The subgroup analysis based on tumor volume according to tumor burdens.

The subgroup analysis based on tumor volume showed that CAR-T was significantly effective in both high tumor burden (WMD = -460.09, 95% CI: -596.38 to -323.80) and low tumor burden (WMD = -527.46, 95% CI: -657.22 to -397.69), but the heterogeneity in the high tumor burden subgroup was minimal (*I²* = 0.0%), while the heterogeneity in the low tumor burden subgroup was high (*I²* = 91.9%) (**Figure 8**). Subgroup analyses were not performed as all mass-related data were low tumor load data.

### Subgroup analysis based on different cell lines

**Figure 9:**
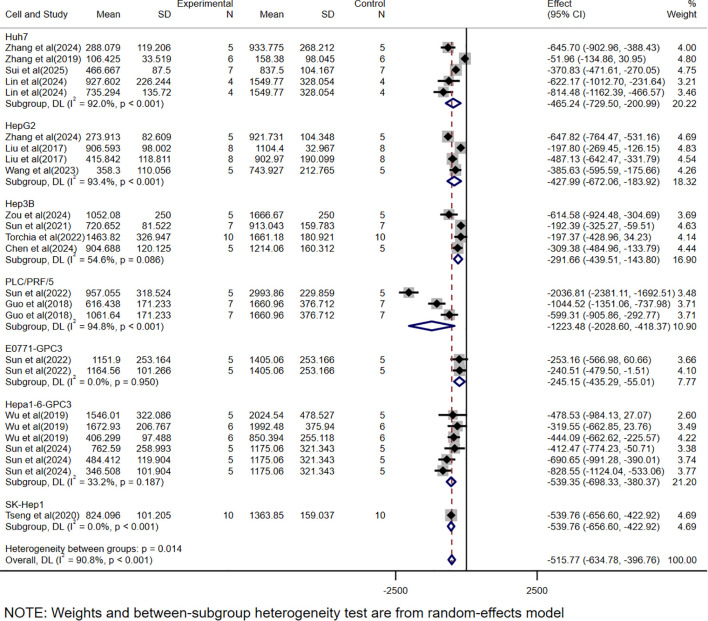
The subgroup analysis based on tumor volume according to cell lines.

**Figure 10:**
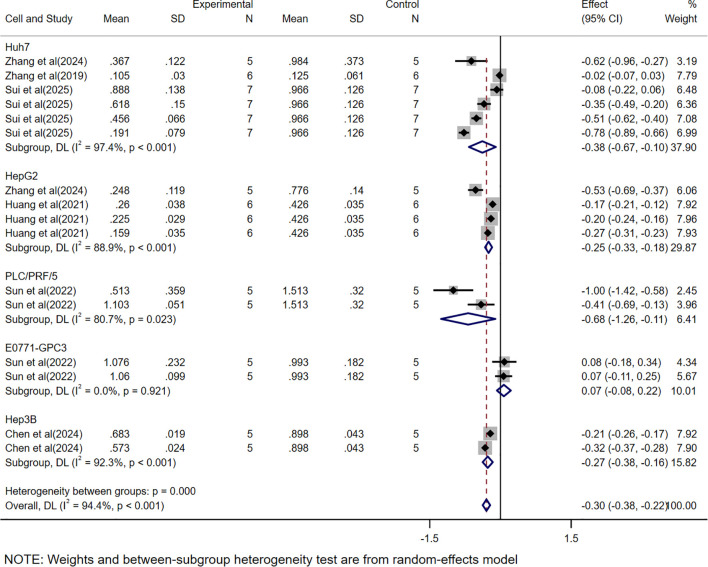
The subgroup analysis based on tumor mass according to cell lines.

We conducted a subgroup analysis based on the cell lines used to construct the mice models. The subgroup analysis based on tumor volume data across different cell lines (**Figure 9**) demonstrated significant efficacy in all cell line subgroups. Among the cell lines included in multiple studies, only the Hep3B cell line (*I²* = 54.6%) and the Hepa1-6-GPC3 cell line (*I²* = 33.2%) exhibited relatively low heterogeneity. Furthermore, the subgroup analysis based on tumor mass across different cell lines (**Figure 10**) revealed significant efficacy in all subgroups except for the E0771-GPC3 cell line (WMD = 0.07, 95% CI: -0.08 to 0.22). Notably, the heterogeneity was consistently high across all cell lines included in multiple studies.

### Subgroup analysis based on different countries

**Figure 11:**
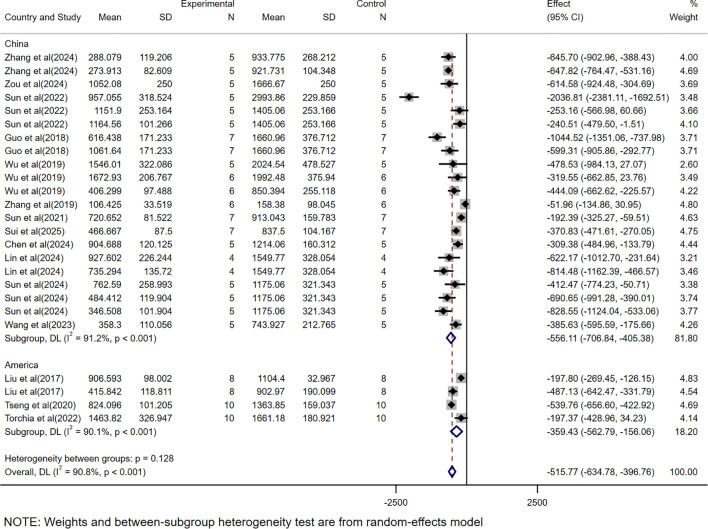
The subgroup analysis based on tumor volume according countries.

The subgroup analysis based on countries showed significant efficacy in studies conducted in China (WMD = -556.11, 95% CI: -706.84 to -405.38) and the United States (WMD = -359.43, 95% CI: -562.79 to -156.06), with high heterogeneity in both (*I²* = 91.2%, 90.1%) (**Figure 11**). Since all mass-related studies were from China, no further subgroup analysis was performed.

### Sensitivity analysis

**Figure 12:**
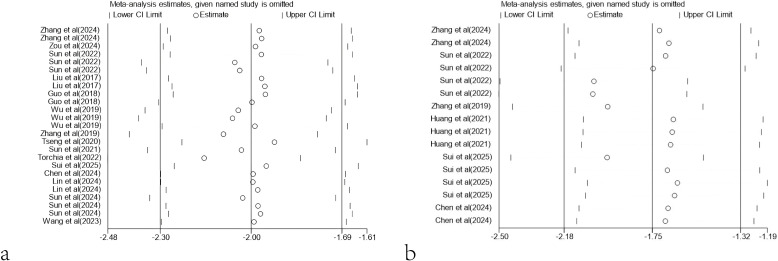
Sensitivity analysis. **(a)** Sensitivity analysis results based on tumor volume. **(b)** Sensitivity analysis results based on tumor mass.

For tumor volume, excluding any of the studies did not significantly affect the overall effect size. However, for tumor mass, excluding the studies by Sun et al. (2022) (WMD = -2.03, 95% CI: -2.49 to -1.58) (WMD = -2.04, 95% CI: -2.50 to -1.58), Zhang et al. (2019) (WMD = -1.97, 95% CI: -2.43 to -1.50) and Sui et al. (2025) (WMD = -1.97, 95% CI: -2.44 to -1.50) would significantly affect the overall effect size (**Figure 12**).

### Bias analysis

**Figure 13:**
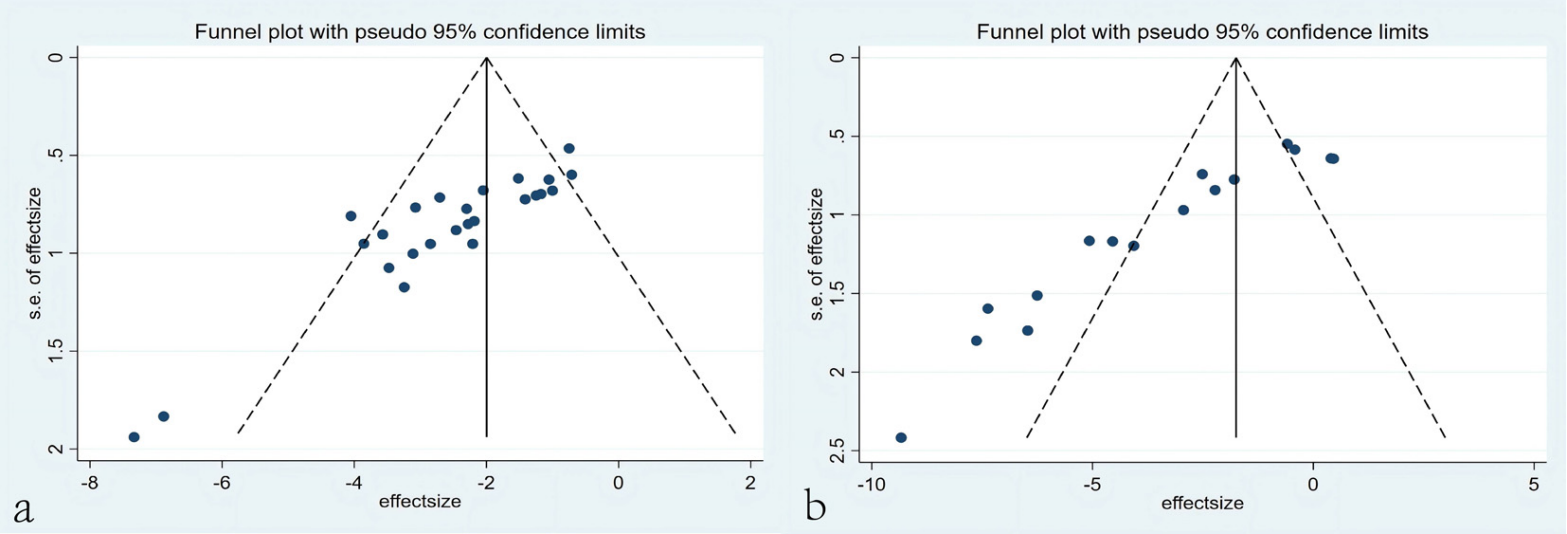
Funnel plots. **(a)** Funnel plots based on tumor volume. **(b)** Funnel plots based on tumor mass.

The funnel plots based on tumor volume and tumor mass data showed good symmetry for tumor volume, indicating that the overall research results were not significantly influenced by publication bias. However, the funnel plot for tumor mass displayed poor symmetry, prompting further investigation through Egger’s regression to explore the presence of publication bias. The results of Egger’s regression revealed significant publication bias for tumor mass data (P = 0.000 < 0.05), suggesting the potential existence of publication bias or omission of small-sample studies (**Figure 13**).

## Discussion

HCC represents one of the leading causes of cancer-related mortality worldwide. In China, HCC exhibits persistently high incidence and mortality rates, with the majority of patients diagnosed at advanced stages, resulting in limited treatment options and poor prognosis. While early-stage HCC patients may achieve favorable survival rates through surgical resection, liver transplantation, or local ablation therapy, advanced-stage patients face limited therapeutic options and exhibit extremely low 5-year survival rates (29). Therefore, the development of novel therapeutic strategies is urgently needed. In recent years, CAR-T therapy has emerged as a novel immunotherapeutic approach. By genetically engineering T cells to specifically recognize and attack tumor cells, CAR-T therapy has demonstrated remarkable efficacy in hematologic malignancies, highlighting its potential for precise targeting and potent tumor cell elimination (30). However, the application of CAR-T therapy in solid tumors, including HCC, remains exploratory, particularly in overcoming immunosuppressive tumor microenvironments and antigen heterogeneity. This study systematically evaluates the therapeutic efficacy of CAR-T therapy in HCC through meta-analysis, aiming to provide robust theoretical foundations for future clinical trials. Additionally, it offers critical insights for optimizing CAR-T cell design, overcoming tumor microenvironment suppression, and exploring combination therapy strategies, thereby advancing the clinical application of CAR-T therapy in HCC treatment.

In this study, we found that CAR-T showed significant therapeutic effect in HCC treatment. By analyzing the data of tumor volume and mass, we observed that the tumor volume and mass were greatly reduced due to the presence of CAR-T. The WMD of tumor volume was -515.77, 95% CI was (-634.78 to -396.76), and the WMD of tumor mass was -0.30, 95% CI was (-0.38 to -0.22). At the same time, the high heterogeneity of the meta-analysis (volume *I²*= 90.8%, mass *I²*= 94.4%) suggests that there may be differences between studies in experimental design, sample characteristics, or intervention protocols, which may affect the stability and consistency of the results. To further investigate the sources of heterogeneity, we conducted a subgroup analysis.

In the subgroup analysis of different mice strains, based on tumor volume data, both immunodeficient mice (WMD = -541.96, 95% CI: -688.79 to -395.13) and immunocompetent mice (WMD = -455.64, 95% CI: -607.79 to -303.48) demonstrated significant therapeutic efficacy, with immunodeficient mice showing superior outcomes. In contrast, analysis based on tumor mass data revealed that immunodeficient mice exhibited significant therapeutic effects (WMD = -0.34, 95% CI: -0.42 to -0.26), whereas immunocompetent mice models did not show statistically significant therapeutic efficacy (WMD = 0.07, 95% CI: -0.08 to 0.22).

This discrepancy may be attributed to the lack of a functional immune system in immunodeficient mice, which reduces the complex immune cell-mediated interactions within the tumor microenvironment. Studies have shown that immunodeficient mice, such as nude or NOD/SCID mice, lack key immune components like T cells, B cells, and NK cells, making tumor growth more dependent on the direct effects of therapeutic agents rather than indirect immune system modulation (31). Conversely, in immunocompetent mice, the intact host immune system may lead to the rapid recognition and clearance of CAR-T cells, thereby diminishing therapeutic efficacy. Additionally, the more complex immune microenvironment in immunocompetent mice may further inhibit the effectiveness of CAR-T cells (32). Therefore, immunodeficient mice models may be more suitable for evaluating the direct antitumor effects of drugs, while immunocompetent mice models better reflect the comprehensive efficacy of drugs in the context of an active immune system.

Notably, the findings of the immunocompetent mice subgroup analysis based on tumor mass data may also be influenced by the limited sample size, as all data were obtained from a single research institution. The restricted sample size and the homogeneity of the data source may limit the generalizability and statistical significance of the results. Thus, future studies should aim to increase sample sizes and incorporate experimental data from diverse sources to validate the efficacy of CAR-T cells in immunocompetent mice and further investigate the underlying mechanisms.

Meanwhile, immunodeficient mice models exhibited high heterogeneity (*I²* = 93,4% and 94.8%), whereas immunocompetent mice models based on volume data showed relatively lower heterogeneity (*I²* = 49.4%). This significant disparity in heterogeneity may stem from the diversity of immunodeficient mice models and the complexity of experimental designs. In contrast, the lower heterogeneity observed in immunocompetent mice models may be attributed to their relatively uniform genetic background and functional immune systems.

To further elucidate and control the high heterogeneity in immunodeficient mice models, we conducted subgroup analyses based on mice strains and found that only C57BL/6 mice, based on tumor volume data, exhibited relatively lower heterogeneity (*I²* = 49.4%). These results further support the hypothesis that immunodeficient mice models exhibit higher heterogeneity. Immunodeficient mice, such as nude, NOD/SCID, or NSG mice, exhibit substantial variations in genetic background, degree of immunodeficiency, and tumor microenvironment characteristics, which may contribute to increased variability in tumor growth and therapeutic responses. For example, NOD/SCID mice lack T and B cells, while NSG mice are further deficient in natural killer (NK) cells and cytokine signaling, differences that may significantly influence tumor growth kinetics and treatment responses (33). Additionally, variations in experimental conditions across laboratories, such as tumor cell inoculation methods, housing environments, and monitoring frequencies, may further exacerbate heterogeneity (34).

In contrast, C57BL/6 mice, as an immunocompetent model, exhibit lower heterogeneity, likely due to their relatively uniform genetic background and functional immune systems. C57BL/6 mice possess intact immune systems that more stably mimic human immune responses, thereby reducing variability in experimental outcomes (35). Furthermore, the widespread use of C57BL/6 mice in tumor research and the accumulation of standardized experimental protocols may also contribute to reduced heterogeneity (36).

However, although C57BL/6 mice exhibit lower heterogeneity, their immunocompetent nature may limit their applicability in certain studies, particularly those requiring the simulation of immunodeficient environments. Therefore, future research may need to further optimize immunodeficient mice models, for example, by constructing more consistent strains through gene-editing technologies or developing standardized experimental protocols to reduce heterogeneity. Additionally, integrating multi-omics analyses and machine learning approaches may help better understand and control heterogeneity in immunodeficient mice models, thereby enhancing experimental reproducibility and clinical translational value (37).

In subgroup analyses based on tumor burden, CAR-T therapy exhibited significant efficacy in both high tumor burden (WMD = -460.09, 95% CI: -596.38 to -323.80) and low tumor burden (WMD = -527.46, 95% CI: -657.22 to -397.69) models, indicating that CAR-T cell therapy has a certain degree of versatility under different tumor burden conditions. Future studies could further explore the specific mechanisms by which tumor burden affects treatment efficacy and optimize combination therapies or CAR-T designs to address the therapeutic needs under different tumor burden conditions. Furthermore, there was high heterogeneity in the low tumor burden subgroup (*I²*=91.9%), which may be attributed to differences in experimental design, treatment protocols, or mice strains in the low tumor burden model. Under low tumor burden conditions, there are fewer tumor targets, which may limit the expansion and persistence of CAR-T cells, thus treatment efficacy could be influenced by various factors, leading to greater variability in the study results. In contrast, the high tumor burden subgroup exhibited minimal heterogeneity (*I²*=0.0%). Given the small sample size in this subgroup, the low heterogeneity may be influenced by insufficient data, failing to capture potential differences. Additionally, under higher tumor burden, the therapeutic effect of CAR-T cell therapy was more consistent, possibly due to the more complex and suppressive tumor microenvironment in high tumor burden models, which enhances the interaction between CAR-T cells and tumors. To further understand the variability in treatment effects under low tumor burden conditions and reduce the bias associated with insufficient sample size, future research should consider standardizing tumor burden classification and optimizing experimental designs to minimize potential confounding factors.

In this study, the low heterogeneity of the Hep3B and Hepa1-6-GPC3 cell lines may suggest that these cell lines exhibit high stability under the experimental conditions. In contrast, the high heterogeneity of the other cell lines likely reflects the variability of these cell lines under different experimental conditions. The Hep3B cell line, a commonly used liver cancer model, has been extensively studied for its biological characteristics. Research has shown that this cell line exhibits high stability under various experimental conditions, with its chromosomal composition remaining consistent across different passages (38). Thus, its low heterogeneity may be attributed to its high experimental reproducibility. The low heterogeneity of the Hepa1-6-GPC3 cell line, a genetically modified cell line, may be related to its specific gene expression pattern. Subgroup analysis in this study indicated significant differences in the response of different cell lines to therapeutic interventions, which may provide important guidance for the experimental design of preclinical studies. When selecting cell lines for experiments, it is essential to carefully consider their heterogeneity and sensitivity to therapeutic interventions. Moreover, for cell lines with high heterogeneity, further optimization of experimental conditions or the incorporation of multiple evaluation metrics may be necessary to enhance the reliability and reproducibility of the experimental results. Similar viewpoints have been supported by other studies, such as in certain lung cancer models, where combining multiple cell lines and evaluation metrics allowed for a more comprehensive assessment of therapeutic intervention outcomes (39).

Meanwhile, subgroup analysis based on country in this study demonstrated that CAR-T cell therapy for HCC in animal experiments showed significant efficacy in both China (WMD = -556.11, 95% CI: -706.84 to -405.38) and the United States (WMD = -359.43, 95% CI: -562.79 to -156.06). However, the heterogeneity was high (*I²*>90%), which may stem from differences in the standardization of experimental procedures, data collection methods, or analytical techniques across research institutions.

In conclusion, the high heterogeneity observed in this study is reasonable and primarily attributable to significant differences between immunodeficient and immunocompetent mice models, variability in experimental designs, variations in tumor burden, and differences in cell line stability. Immunodeficient models exhibit greater heterogeneity due to their diverse genetic backgrounds and varying degrees of immunodeficiency, whereas immunocompetent models demonstrate lower heterogeneity owing to their uniform genetic and immune characteristics. Additionally, the complexity of the tumor microenvironment, variations in cell lines, and differences in experimental conditions further amplify the variability in outcomes. Collectively, these factors justify the high heterogeneity as both reasonable and expected in this study, while also highlighting the need for standardized protocols and larger sample sizes in future research.

In addition, sensitivity analysis also showed that the results of meta-analysis based on tumor volume and tumor mass were robust. Although the exclusion of specific datasets (such as those from Sun et al., 2022) had some impact on the statistical significance of tumor mass, the overall conclusions remained consistent and reliable. When the data from Sun et al. (2022) were excluded, the combined effect size reached the maximum, indicating that the results of this study had a negative impact on the overall effect size. Sun et al. (2022) found no significant difference in efficacy between the 28ζCAR-T and BBζCAR-T treatment groups and the control group. This result may indicate that 28ζCAR-T and BBζCAR-T have a limited therapeutic effect under experimental conditions. Possible reasons for this result include the unsatisfactory response to 28ζCAR-T and BBζCAR-T in immunocompetent mice models constructed by E0771-GPC3 cells, problems with the quality or dose of 28ζCAR-T and BBζCAR-T, or changes in experimental conditions. It is also possible that the use of 28ζCAR-T and BBζCAR-T alone is not sufficient and may need to be combined with other treatments to improve efficacy.

Finally, we conducted a bias detection analysis on tumor volume and mass data, finding that tumor volume data exhibited minimal bias, while tumor mass data showed greater bias. The bias in tumor mass may stem from differences in measurement methods after tumor excision, particularly operational errors during mass measurement, such as moisture loss, tissue damage, or interference from non-tumor components. This is especially true when tumor tissue has not been completely excised or when tissue damage occurs during excision, which can lead to fluctuations and instability in mass data. These factors render tumor mass measurement unstable, leading to greater variability in the data. Compared to volume measurements, the complexity of tumor mass measurement and its high demands for operational precision make it more prone to significant bias in experiments. In addition to measurement errors, sample size and the heterogeneity of study designs may also exacerbate bias, particularly in studies with small sample sizes and inconsistent experimental procedures. Furthermore, publication bias and reporting bias may result in certain mass data being underreported or excluded from the meta-analysis. To reduce bias, future studies should focus on more standardized tumor mass measurement methods, improve the consistency of experimental designs, and increase sample sizes, thereby enhancing data reliability and the accuracy of meta-analyses.

At the same time, the limitations of this study need to be carefully considered, as they may affect the interpretation and application of the results.

First, this study did not perform subgroup analyses of CAR-T cell target antigens, dosing regimens, or co-administration therapies. Among them, the diversity of CAR-T cell target antigens is an important factor, and there are large differences in the tumor antigens targeted by CAR-T cells in different studies, and the number of CAR-T studies targeting a particular target antigen is quite limited, so we were unable to perform subgroup analyses to assess the relative advantages of CAR-T cells with different target antigens in HCC treatment. Second, most of the studies used intravenous injection as the mode of CAR-T cell delivery, with fewer studies of intra-tumoral injection, thus again preventing relevant subgroup analyses. In addition, due to the lack of data, we were also unable to perform subgroup analyses for other factors such as co-administration therapies, thus limiting in-depth comparisons of different treatment strategies.

The asymmetry in the funnel plot for tumor mass suggests the potential presence of publication bias, which may arise from the selective publication of studies with positive outcomes (i.e., those demonstrating significant tumor mass reduction with CAR-T therapy) while neglecting studies with negative or null results. This bias could lead to an overestimation of the therapeutic efficacy of CAR-T therapy, causing the overall effect size to deviate from the true value. Furthermore, publication bias may exacerbate heterogeneity among studies (I²=91.4%), further undermining the robustness of the conclusions. Sensitivity analysis revealed that the exclusion of certain studies (e.g., Sun et al., 2022) resulted in significant changes in the effect size, indicating that these studies had a substantial impact on the overall findings. If these studies themselves were influenced by publication bias, the reliability of the conclusions would be further compromised. Therefore, although the meta-analysis demonstrated a significant reduction in tumor mass with CAR-T therapy, the conclusions should be interpreted with caution due to potential publication bias and high heterogeneity. Future research should focus on minimizing publication bias and enhancing data transparency to improve the reliability of the conclusions.

Although this study systematically evaluated the therapeutic efficacy of CAR-T therapy in HCC through meta-analysis, it is crucial to acknowledge that these findings are predominantly derived from preclinical trials and lack robust clinical validation. Although preclinical studies offer valuable preliminary evidence regarding CAR-T therapy’s potential, their translational application to human trials presents significant challenges, particularly in addressing tumor heterogeneity and microenvironmental immunosuppressive complexity.

In preclinical models, CAR-T therapy has demonstrated significant antitumor efficacy in immunodeficient mice, particularly in reducing tumor volume and mass. However, its efficacy is relatively limited in immunocompetent mice models, potentially due to host immune rejection and tumor microenvironment-mediated immunosuppression. In humans, the tumor microenvironment is typically more complex, comprising various immunosuppressive cells and factors (e.g., regulatory T cells, tumor-associated macrophages, TGF-β, etc.) (40), which may further compromise CAR-T cell efficacy. Therefore, although preclinical findings are promising, CAR-T therapy may require combination with other immunomodulatory strategies (e.g., PD-1/PD-L1 inhibitors, CTLA-4 inhibitors, etc.) in human trials to overcome immunosuppressive microenvironmental limitations (41).

Animal models are indispensable in preclinical studies of CAR-T cell therapies, but substantial discrepancies between these models and human clinical applications may hinder the direct translation of research findings. Firstly, animal models (e.g., immunodeficient mice) lack a fully functional immune system, thus failing to accurately replicate the complex immunosuppressive microenvironment characteristic of human tumors. Secondly, human HCC exhibits remarkable heterogeneity, characterized by diverse genetic mutations (e.g., TP53, CTNNB1) and epigenetic modifications, whereas animal models relying on single cell lines or genetically engineered mice cannot adequately recapitulate this complexity (42). Moreover, animal experiments are typically conducted under highly controlled laboratory conditions that fail to account for the multifaceted environmental factors influencing human physiology, potentially limiting the clinical relevance of the findings. These limitations indicate that while animal models offer valuable insights for preliminary assessment of CAR-T therapies, their findings necessitate rigorous validation and optimization through human clinical trials.

Notably, several clinical trials investigating CAR-T therapy for HCC are currently underway. For instance, CAR-T cell therapies targeting HCC-associated antigens such as GPC3 and AFP have demonstrated acceptable safety profiles and preliminary efficacy in early-phase clinical trials (43). Additionally, some trials are exploring combination strategies of CAR-T cells with immune checkpoint inhibitors, targeted therapies, or radiotherapy, aiming to overcome tumor microenvironment-mediated immunosuppression while enhancing CAR-T cell persistence and antitumor efficacy (44, 45). These clinical trials have not only generated valuable data supporting CAR-T therapy applications in HCC but also provided critical insights for optimizing CAR-T cell design and combination therapy strategies.

Furthermore, additional limitations of preclinical studies include relatively small sample sizes, highly standardized experimental conditions, and insufficient assessment of long-term safety and efficacy. These factors may constrain the generalizability of the research findings. To address these limitations, future studies should incorporate larger sample sizes and validate CAR-T therapy efficacy through multicenter, multisample experimental designs.

Finally, potential biases may exist in the data extraction process, particularly when using WebPlotDigitizer software to extract data from graphical representations. Factors such as image resolution, axis clarity, and data point discernibility may affect the accuracy of data extraction. For instance, low-resolution images or unclear axes may lead to errors in data extraction, and overlapping or densely clustered data points may hinder the accurate differentiation of individual points. Additionally, manual intervention by the operator in selecting data points and calibrating axes could introduce human error, thereby impacting the reliability of the results. Although we have validated the robustness of the results through sensitivity and subgroup analyses, these potential extraction biases require cautious interpretation. To minimize such biases, future studies should prioritize obtaining raw data directly from original publications or by contacting the authors for comprehensive datasets. Moreover, using high-resolution graphs with clearly labeled axes and employing multiple independent operators with consistency checks are recommended to reduce operator-dependent errors.

In summary, this study systematically assessed the therapeutic efficacy of CAR-T therapy in HCC through meta-analysis, demonstrating its substantial anti-tumor potential in preclinical investigations. Despite the presence of certain heterogeneity and potential biases, the overall findings indicate that CAR-T therapy significantly reduces tumor volume and mass. However, these findings are predominantly derived from animal studies and require further validation through large-scale clinical trials. Future research should focus on optimizing experimental designs, investigating the impact of various cell lines and mice strains on therapeutic outcomes, and advancing CAR-T cell design optimization, tumor microenvironment modulation, and combined therapeutic strategy development to facilitate the clinical translation of CAR-T therapies for HCC treatment. Additionally, comprehensive long-term safety assessments and multicenter clinical trials will establish a robust foundation for the widespread implementation of CAR-T therapy in HCC management.

## Data Availability

The original contributions presented in the study are included in the article/supplementary material. Further inquiries can be directed to the corresponding authors.

## References

[1] Nishiguchi S Kuroki T Nakatani S Morimoto H Takeda T Nakajima S . Randomised trial of effects of interferon-alpha on incidence of hepatocellular carcinoma in chronic active hepatitis C with cirrhosis. Lancet. (1995) 346:1051–5. doi: 10.1016/S0140-6736(95)91739-X 7564784

[2] Yuan ZG Ye SL . Systemic therapeutic strategies for hepatocellular carcinoma: current status and prospects. Zhonghua gan zang bing za zhi = Zhonghua ganzangbing zazhi = Chin J hepatology. (2024) 32:565–71. doi: 10.3760/cma.j.cn501113-20240412-00200 PMC12899070 38964901

[3] Reig M Forner A Rimola J Ferrer-Fàbrega J Burrel M Garcia-Criado Á . BCLC strategy for prognosis prediction and treatment recommendation: The 2022 update. J Hepatol. (2022) 76:681–93. doi: 10.1016/j.jhep.2021.11.018 PMC8866082 34801630

[4] Tao Z Chyra Z Kotulová J Celichowski P Mihályová J Charvátová S . Impact of T cell characteristics on CAR-T cell therapy in hematological Malignancies. Blood Cancer J. (2024) 14:213. doi: 10.1038/s41408-024-01193-6 39627220 PMC11615218

[5] Maude S Laetsch T Buechner J Rives S Boyer M Bittencourt H . Tisagenlecleucel in children and young adults with B-cell lymphoblastic leukemia. N Engl J Med. (2018) 378:439–48. doi: 10.1056/NEJMoa1709866 PMC5996391 29385370

[6] Swan D Madduri D Hocking J . CAR-T cell therapy in Multiple Myeloma: current status and future challenges. Blood Cancer J. (2024) 14:206. doi: 10.1038/s41408-024-01191-8 39592597 PMC11599389

[7] Zheng H Xian H Zhang W Lu C Pan R Liu H . BCMA-targeted therapies for multiple myeloma: latest updates from 2024 ASH annual meeting. J Hematol Oncol. (2025) 18:23. doi: 10.1186/s13045-025-01675-5 PMC11872297 40025529

[8] Zhou Y Wei S Xu M Wu X Dou W Li H . CAR-T cell therapy for hepatocellular carcinoma: current trends and challenges. Front Immunol. (2024) 15:1489649. doi: 10.3389/fimmu.2024.1489649 39569202 PMC11576447

[9] Du G Dou C Sun P Wang S Liu J Ma L . Regulatory T cells and immune escape in HCC: understanding the tumor microenvironment and advancing CAR-T cell therapy. Front Immunol. (2024) 15:1431211. doi: 10.3389/fimmu.2024.1431211 39136031 PMC11317284

[10] Batra SA Rathi P Guo L Courtney AN Fleurence J Balzeau J . Glypican-3-specific CAR T cells coexpressing IL15 and IL21 have superior expansion and antitumor activity against hepatocellular carcinoma. Cancer Immunol Res. (2020) 8:309–20. doi: 10.1158/2326-6066.CIR-19-0293 PMC10765595 31953246

[11] Zhou L Li Y Zheng D Zheng Y Cui Y Qin L . Bispecific CAR-T cells targeting FAP and GPC3 have the potential to treat hepatocellular carcinoma. Mol Therapy: Oncol. (2024) 32:200817. doi: 10.1016/j.omton.2024.200817 PMC11179089 38882528

[12] Liu H Xu Y Xiang J Long L Green S Yang Z . Targeting alpha-fetoprotein (AFP)-MHC complex with CAR T-cell therapy for liver cancer. Clin Cancer Res. (2017) 23:478–88. doi: 10.1158/1078-0432.CCR-16-1203 27535982

[13] Liu F Qian Y . The role of CD133 in hepatocellular carcinoma. Cancer Biol Ther. (2021) 22:291–300. doi: 10.1080/15384047.2021.1916381 33899676 PMC8183534

[14] Chen S Gong F Liu S Xie Y Ye X Lin X . IL-21- and CXCL9-engineered GPC3-specific CAR-T cells combined with PD-1 blockade enhance cytotoxic activities against hepatocellular carcinoma. Clin Exp Med. (2024) 24:204. doi: 10.1007/s10238-024-01473-2 PMC11358300 39196390

[15] Giardino Torchia ML Gilbreth R Merlino A Sult E Monks N Chesebrough J . Rational design of chimeric antigen receptor T cells against glypican 3 decouples toxicity from therapeutic efficacy. Cytotherapy. (2022) 24(7):720–32. doi: 10.1016/j.jcyt.2022.03.008 35570170

[16] Guo X Jiang H Shi B Zhou M Zhang H Shi Z . Disruption of PD-1 enhanced the anti-tumor activity of chimeric antigen receptor T cells against hepatocellular carcinoma. Front Pharmacol. (2018) 9. doi: 10.3389/fphar.2018.01118 PMC6174208 30327605

[17] Huang X Guo J Li T Jia L Tang X Zhu J . c-Met-targeted chimeric antigen receptor T cells inhibit hepatocellular carcinoma cells *in vitro* and *in vivo* . J Biomed Res. (2021) 36:10–21. doi: 10.7555/JBR.35.20200207 35403606 PMC8894281

[18] Lin K Xia B Wang X He X Zhou M Lin Y . Development of nanobodies targeting hepatocellular carcinoma and application of nanobody-based CAR-T technology. J Trans Med. (2024) 22:349. doi: 10.1186/s12967-024-05159-x PMC11015683 38610029

[19] Sui M Liu T Song X Li J Ding H Liu Y . The molecular receptor NKBB enhances the persistence and anti-hepatocellular carcinoma activity of GPC3 CAR-T cells. Pharmacol Res. (2025) 212:107619. doi: 10.1016/j.phrs.2025.107619 39842473

[20] Sun L Gao F Gao Z Ao L Li N Ma S . Shed antigen-induced blocking effect on CAR-T cells targeting Glypican-3 in Hepatocellular Carcinoma. J Immunother Cancer. (2021) 9:e001875. doi: 10.1136/jitc-2020-001875 33833049 PMC8039282

[21] Sun R Liu Y-F Sun Y Zhou M Wang Y Shi B . GPC3-targeted CAR-T cells expressing GLUT1 or AGK exhibit enhanced antitumor activity against hepatocellular carcinoma. Acta Pharmacologica Sinica. (2024) 45:1937–50. doi: 10.1038/s41401-024-01287-8 PMC11336244 38750075

[22] Sun Y Dong Y Sun R Liu Y Wang Y Luo H . Chimeric anti-GPC3 sFv-CD3ϵ receptor-modified T cells with IL7 co-expression for the treatment of solid tumors. Mol Ther Oncolytics. (2022) 25:160–73. doi: 10.1016/j.omto.2022.04.003 PMC9065615 35572194

[23] Tseng H-c Xiong W Badeti S Yang Y Ma M Liu T . Efficacy of anti-CD147 chimeric antigen receptors targeting hepatocellular carcinoma. Nat Commun. (2020) 11:4810. doi: 10.1038/s41467-020-18444-2 PMC7511348 32968061

[24] Wang X Lai Z Pang Y Sun Q Yang W Wang W . PD-1 blocking strategy for enhancing the anti-tumor effect of CAR T cells targeted CD105. Heliyon. (2023) 9:e12688. doi: 10.1016/j.heliyon.2022.e12688 36685461 PMC9849980

[25] Wu X Luo H Shi B Di S Sun R Su J . Combined antitumor effects of sorafenib and GPC3-CAR T cells in mouse models of hepatocellular carcinoma. Mol Ther. (2019) 27:1483–94. doi: 10.1016/j.ymthe.2019.04.020 PMC6697347 31078430

[26] Zhang Q Su C Luo Y Zheng F Liang C-L Chen Y . Astragalus polysaccharide enhances antitumoral effects of chimeric antigen receptor- engineered (CAR) T cells by increasing CD122+CXCR3+PD-1- memory T cells. BioMed Pharmacother. (2024) 179:117401. doi: 10.1016/j.biopha.2024.117401 39243425

[27] Zhang R-Y Wei D Liu Z-K Yong Y-L Wei W Zhang Z-Y . Doxycycline inducible chimeric antigen receptor T cells targeting CD147 for hepatocellular carcinoma therapy. Front Cell Dev Biol. (2019) 7. doi: 10.3389/fcell.2019.00233 PMC6798074 31681766

[28] Zou Q Liao K Li G Huang X Zheng Y Yang G-H . Photo-metallo-immunotherapy: fabricating chromium-based nanocomposites to enhance CAR-T cell infiltration and cytotoxicity against solid tumors. Adv Mater. (2024) 37(2):2407425. doi: 10.1002/adma.202407425 PMC11733712 38899741

[29] Cillo U Gringeri E D’Amico FE Lanari J Furlanetto A Vitale A . Hepatocellular carcinoma: Revising the surgical approach in light of the concept of multiparametric therapeutic hierarchy. Dig Liver Dis. (2025) S1590-8658(24)01123–X. doi: 10.1016/j.dld.2024.12.003 39828438

[30] Caël B Bôle-Richard E Garnache Ottou F Aubin F . Chimeric antigen receptor-modified T-cell therapy: Recent updates and challenges in autoimmune diseases. J Allergy Clin Immunol. (2025) 155:688–700. doi: 10.1016/j.jaci.2024.12.1066 39675682

[31] Ahmed EN Cutmore LC Marshall JF . Syngeneic mouse models for pre-clinical evaluation of CAR T cells. Cancers. (2024) 16:3186. doi: 10.3390/cancers16183186 39335157 PMC11430534

[32] Ramos A Koch CE Liu-Lupo Y Hellinger RD Kyung T Abbott KL . Leukemia-intrinsic determinants of CAR-T response revealed by iterative *in vivo* genome-wide CRISPR screening. Nat Commun. (2023) 14:8048. doi: 10.1038/s41467-023-43790-2 38052854 PMC10698189

[33] Chen J Liao S Xiao Z Pan Q Wang X Shen K . The development and improvement of immunodeficient mice and humanized immune system mouse models. Front Immunol. (2022) 13:1007579. doi: 10.3389/fimmu.2022.1007579 36341323 PMC9626807

[34] Gengenbacher N Singhal M Augustin HG . Preclinical mouse solid tumour models: status quo, challenges and perspectives. Nat Rev Cancer. (2017) 17:751–65. doi: 10.1038/nrc.2017.92 29077691

[35] Bergsagel PL Chesi M . Immunocompetent mouse models of multiple myeloma. Semin Hematol. (2024) (2025) 62(1):50–7. doi: 10.1053/j.seminhematol.2024.11.003 PMC11911088 39674742

[36] Ayala EV Cunha G Azevedo MA Calderón M Jiménez J Venuto AP . C57BL/6 α1,3-galactosyltransferase knockout mouse (α-galT-KO) as an animal model for experimental chagas disease. ACS Infect Dis. (2020) 6:1807–15. doi: 10.1021/acsinfecdis.0c00061 32374586

[37] Satija N Patel F Schmidt G Doanman DV Kapoor M La Porte A . Tracking HIV persistence across T cell lineages during early ART-treated HIV-1-infection using a reservoir-marking humanized mouse model. Nat Commun. (2025) 16:2233. doi: 10.1038/s41467-025-57368-7 40044684 PMC11883074

[38] Wang M Weng S Li C Jiang Y Qian X Xu P . Proteomic overview of hepatocellular carcinoma cell lines and generation of the spectral library. Sci Data. (2022) 9:732. doi: 10.1038/s41597-022-01845-x 36446815 PMC9708666

[39] Giron-Michel J Padelli M Oberlin E Guenou H Duclos-Vallée J-C . State-of-the-art liver cancer organoids: modeling cancer stem cell heterogeneity for personalized treatment. BioDrugs. (2025) 39(2):237–60. doi: 10.1007/s40259-024-00702-0 PMC11906529 39826071

[40] Kong Y Li J Zhao X Wu Y Chen L . CAR-T cell therapy: developments, challenges and expanded applications from cancer to autoimmunity. Front Immunol. (2025) 15:1519671. doi: 10.3389/fimmu.2024.1519671 39850899 PMC11754230

[41] Andreu-Saumell I Rodriguez-Garcia A Mühlgrabner V Gimenez-Alejandre M Marzal B Castellsagué J . CAR affinity modulates the sensitivity of CAR-T cells to PD-1/PD-L1-mediated inhibition. Nat Commun. (2024) 15:3552. doi: 10.1038/s41467-024-47799-z 38670972 PMC11053011

[42] Li L Wang H . Heterogeneity of liver cancer and personalized therapy. Cancer Letters. (2016) 379:191–7. doi: 10.1016/j.canlet.2015.07.018 26213370

[43] Li M Chen T Huang R Cen Y Zhao F Fan R . Chimeric antigen receptor-T cells targeting AFP-GPC3 mediate increased antitumor efficacy in hepatocellular carcinoma. Arab J Gastroenterol. (2025) 26(1):84–93. doi: 10.1016/j.ajg.2024.12.002 39757079

[44] Ma X Zhang W Zeng M Asavasupreechar T Kang S Li Y . Systemic tumor regression with synergy therapy: radiotherapy and CAR-T. Cell Death Discovery. (2024) 10:479. doi: 10.1038/s41420-024-02245-3 PMC11584735 39578426

[45] Xia X Yang Z Lu Q Liu Z Wang L Du J . Reshaping the tumor immune microenvironment to improve CAR-T cell-based cancer immunotherapy. Mol Cancer. (2024) 23:175. doi: 10.1186/s12943-024-02079-8 39187850 PMC11346058

